# The GPR30 agonist G-1 promotes hair growth via Wnt/Hedgehog signaling in mice

**DOI:** 10.3389/fphar.2025.1570922

**Published:** 2025-07-11

**Authors:** Mayu Yamano, Yuto Yamanaka, Shota Nishikawa, Yuki Masujima, Ryuji Ohue-Kitano, Ikuo Kimura

**Affiliations:** ^1^ Department of Molecular Endocrinology, Graduate School of Pharmaceutical Sciences, Kyoto University, Kyoto, Japan; ^2^ Laboratory of Molecular Neurobiology, Graduate School of Biostudies, Kyoto University, Kyoto, Japan; ^3^ Department of Biological and Environmental Chemistry, Kindai University, Fukuoka, Japan; ^4^ Japan Agency for Medical Research and Development–Core Research for Evolutional Science and Technology (AMED–CREST), Japan Agency for Medical Research and Development, Tokyo, Japan

**Keywords:** GPR30, estrogen, hair growth, G-1, Wnt/Hedgehog signaling

## Abstract

**Background:**

GPR30 is a membrane-associated receptor involved in rapid, non-genomic estrogen signaling. Estrogen significantly influences hair growth and susceptibility to hair loss, with differences primarily driven by hormonal factors. While estrogen’s role in regulating hair follicle cycling is recognized, its precise molecular mechanisms remain unclear. This study investigates the role of GPR30 in hair follicle biology and evaluates its potential as a therapeutic target for estrogen-mediated hair loss disorders.

**Methods:**

The GPR30 selective agonist G-1 was administrated to female *Gpr30*-deficient mice with a C57BL/6J background and human hair follicle dermal papilla cell, and the effects on hair growth and the molecular signaling were evaluated.

**Results:**

We demonstrate that GPR30 is abundantly expressed in mouse skin, particularly during the anagen phase of the hair follicle cycle, implicating it in hair growth regulation. Activation of GPR30 using the selective agonist G-1 in mouse skin and human dermal papilla cells significantly upregulated Wnt/Hedgehog signaling, which are key pathways promoting hair growth. These effects were absent in *Gpr30*-deficient mice or in those administered a GPR30 antagonist, confirming the essential role of GPR30 in estrogen-mediated regulation of hair follicle activity.

**Conclusion:**

Our findings underscore the importance of GPR30 in modulating hair growth and suggest that GPR30, along with its selective agonists, holds promise as a novel therapeutic target for treating hair loss disorders and other estrogen-responsive conditions.

## Introduction

Hair growth is a complex process regulated by genetic, hormonal, and environmental factors. The hair follicle undergoes cyclic phases of rest (telogen), growth (anagen), and regression (catagen), which are controlled by signaling pathways such as Wnt/β-catenin (Fuchs et al., 2001; Clevers et al., 2014; Choi, 2020). The Wnt/β-catenin pathway plays a critical role in hair follicle development, regeneration, and cycling by activating stem cells and interacting with other signaling pathways, including Hedgehog, BMP, and FGF (Colin-Pierre et al., 2022; Bai et al., 2019). Activation of Wnt and Hedgehog signaling has been shown to promote hair follicle neogenesis and regeneration, whereas its inhibition is linked to alopecia. Sex differences significantly influence hair growth patterns and susceptibility to hair loss, primarily due to hormonal factors such as estrogen and androgens (Stenn and Paus, 2001; Grymowicz et al., 2020; Zouboulis et al., 2007; Ohnemus et al., 2006; Conrad and Paus, 2004). Nuclear estrogen receptors (ERα and ERβ) are highly expressed in hair follicle cells, suggesting that estrogens exert a direct regulatory effect on follicle dynamics (Thornton et al., 2006; Thornton et al., 2003). However, despite advances in understanding estrogen’s role in hair follicle biology, the precise molecular mechanisms underlying its effects on hair growth remain incompletely understood.

GPR30, also known as G protein-coupled estrogen receptor 1, is a membrane-associated receptor mediating rapid non-genomic estrogen signaling (Olde and Leeb-Lundberg, 2009; Prossnitz and Barton, 2023). Unlike classical nuclear estrogen receptors, GPR30 is located in the cell membrane and cytoplasm, where it activates intracellular signaling cascades such as cAMP production, calcium mobilization, and ERK1/2 activation (Prossnitz and Arterburn, 2015; Natale et al., 2016; Wang et al., 2023; Kuo et al., 2010). These signaling events regulate key cellular processes, including proliferation and apoptosis, depending on the tissue context (Sharma et al., 2020; Gonzalez de Valdivia et al., 2017; Dennis et al., 2009). Although GPR30 has been studied in various tissues, its role in hair follicle biology remains unexplored.

Given the lack of research on non-classical estrogen receptors in hair growth regulation, this study aimed to investigate the molecular mechanisms by which GPR30 influences hair follicle activity. Using *Gpr30*-deficient mice and the selective agonist G-1, we explored GPR30’s role in regulating hair growth and assessed its potential as a therapeutic target for hair loss disorders and other estrogen-responsive conditions.

## Materials and methods

### Animal study

Male and female C57BL/6J wild-type and *Gpr30*-deficient mice were housed under a 12-h light–dark cycle and fed regular chow (CE-2, CLEA, Tokyo, Japan). *Gpr30*-deficient mice were generated on a C57BL/6J background using the CRISPR/Cas9 system. Six-week-old female wild-type and *Gpr30*-deficient mice were used for hair growth and cycle experiments. All experimental procedures involving mice adhered to the protocols approved by the Kyoto University Animal Experimentation Committee (Lif-K21020), and efforts were made to minimize animal suffering.

### RNA extraction and quantitative reverse transcription-polymerase chain reaction (qRT-PCR)

Total RNA was extracted from mouse skin using an RNeasy Mini Kit (Qiagen, Hilden, Germany) and RNAiso Plus (Takara, Shiga, Japan). Complementary DNA (cDNA) was synthesized using Moloney murine leukemia virus reverse transcriptase (Invitrogen, Carlsbad, CA, United States). qRT-PCR was performed with SYBR Premix Ex Taq II (Takara) on StepOne™ and QuantStudio™ 1 real-time PCR systems (Applied Biosystems, CA, United States), following previously described protocols (Kimura et al., 2020). The primer sequences for targeted mouse genes were as follows: *18S*, 5ʹ-CTC​AAC​ACG​GGA​AAC​CTC​AC-3ʹ (forward) and 5ʹ-AGA​CAA​ATC​GCT​CCA​CCA​AC-3ʹ (reverse); *Gpr30*, 5′-GGG​TGC​CAG​GAC​AAT​GAA​ATA​CTC-3ʹ (forward) and 5ʹ- ATC​CGC​ACA​TGA​CAG​GTT​TAT​TGA-3ʹ (reverse); *Rspo2*, 5′-TTG​CAT​AGA​GGC​CGC​TGC​TTT-3′ (forward) and 5′-CTG​GTC​AGA​GGA​TCA​GGA​ATG-3′ (reverse); *Lef1*, 5′- TGA​GTG​CAC​GCT​AAA​GGA​GA-3′ (forward) and 5′-CTG​ACC​AGC​CTG​GAT​AAA​GC-3′ (reverse); *Gli1*, 5′- GGA​AGT​CCT​ATT​CAC​GCC​TTG​A-3′ (forward) and 5′- CAA​CCT​TCT​TGC​TCA​CAC​ATG​TAA​G-3′ (reverse); *Ptch1*, 5′- CTC​TGG​AGC​AGA​TTT​CCA​AGG-3′ (forward) and 5′- TGC​CGC​AGT​TCT​TTT​GAA​TG-3′ (reverse). The primer sequences for targeted human genes were as follows: *RPLPO*, 5ʹ-GAA​GCC​ACG​CTG​CTG​AAC​A-3ʹ (forward) and 5ʹ-CTG​GCA​ACA​TTG​CGG​ACA-3ʹ (reverse); *RSPO2*, 5′-TGG​CTC​AGT​GTG​TGC​TGA​GAG​AAT-3′ (forward) and 5′-AAG​GTC​ACG​AGT​GAG​TAG​CGC​ATT-3′ (reverse); *RSPO3*, 5′-TGC​ACT​GTG​AGG​TCA​GTG​AAT​GGA-3′ (forward) and 5′-AGG​TTA​CCC​TTT​GCT​GAA​GGA​TGC-3′ (reverse); *LEF1*, 5′-ATG​CCA​CCT​TCT​GCC​AAG​AA-3′ (forward) and 5′-ATC​ACA​CCC​GTC​ACA​CAT​CC-3′ (reverse); *GLI1*, 5′-AGC​CTT​CAG​CAA​TGC​CAG​TGA​C-3′ (forward) and 5′-GTC​AGG​ACC​ATG​CAC​TGT​CTT​G-3′ (reverse); *PTCH1*, 5′-GCT​GCA​CTA​CTT​CAG​AGA​CTG​G-3′ (forward) and 5′-CAC​CAG​GAG​TTT​GTA​GGC​AAG​G-3′ (reverse); *CCND1*, 5′-TCT​ACA​CCG​ACA​ACT​CCA​TCC​G-3′ (forward) and 5′-TCT​GGC​ATT​TTG​GAG​AGG​AAG​TG-3′ (reverse).

### Hair growth and cycle experiments

To synchronize the hair cycle, the dorsal region of each mouse was shaved using electric clippers and depilatory cream to induce telogen-to-anagen transition. The shaved area was carefully monitored to ensure complete hair removal without skin damage. Solutions of 17-β-estradiol (FUJIFILM Wako, Osaka, Japan), G-1 (Cayman Chemical, MI, United States), or minoxidil (Tokyo Chemical Industry, Tokyo, Japan) were prepared in ethanol containing 2% DMSO (FUJIFILM Wako) for uniform delivery. Two days after shaving, 100 µL of the solution was applied daily to the shaved dorsal area for 24 days. The control group received vehicle alone under the same conditions. Solutions were applied using a pipette and spread evenly with a sterile applicator to ensure complete coverage. Hair growth was monitored daily, and the results were evaluated at the end of the treatment period. Photographs documented progress, and pigmentation, hair density, and hair length were assessed. Hair growth stages were classified as telogen (no visible growth), early anagen (pigmentation and short hair emergence), and late anagen (fully extended hair). These experiments were conducted as independent application trials using three biological replicates.

### RNA sequencing

RNA was extracted from the back skin of wild-type and *Gpr30*-deficient mice using RNAiso Plus (Takara) and purified with an RNeasy Mini Kit (Qiagen). cDNA libraries were prepared for RNA sequencing using the NEBNext^®^ Ultra™ II Directional RNA Library Prep Kit (Illumina, CA, United States) and NEBNext Multiplex Oligos for Illumina (Dual Index Primers Set 1). Sequencing was performed on an Illumina NovaSeq 6000 platform, generating approximately four gigabases of paired-end reads per sample, with a read length of 100 bp. Raw sequencing data were preprocessed using Trimmomatic to remove adapter sequences and low-quality reads (Bolger et al., 2014). The quality of trimmed reads was assessed with FastQC (Cock et al., 2010). Reads were then aligned to the mouse reference genome (mm10) using HISAT2 (Kim et al., 2015), which incorporates the Bowtie2 aligner (Langmead and Salzberg, 2012). The aligned reads were assembled with StringTie (Pertea et al., 2015). Raw read counts were normalized using the relative log expression (RLE) method, and differentially expressed genes (DEGs) were identified using DESeq2. Fold changes were calculated using the nbinomWald test. DEGs were defined as having a false discovery rate (FDR)-adjusted p-value < 0.05 (Benjamini-Hochberg correction) and an absolute |log_2_ (fold change)| > 0.5. Gene Set Enrichment Analysis (GSEA) was performed using the Kyoto Encyclopedia of Genes and Genomes (KEGG) database (http://www.genome.jp/kegg/) and included Gene Ontology (GO) terms related to molecular functions, biological processes, cellular components, and pathways.

### Primary culture of human hair follicle dermal papilla cells

Human hair follicle dermal papilla cells (hDPCs) were obtained from PromoCell (Heidelberg, Germany) and cultured in follicle dermal papilla cell growth medium (Promocell) supplemented with a growth medium Supplement Pack (Promocell). Cultures were maintained at 37°C in a humidified incubator with 5% CO2. hDPCs were seeded into 6-well plates, and G-1 (100 nM) or G-36 (10 μM) were added 24 h later. RNA and proteins were collected 24 h post-treatment for subsequent gene and protein expression analysis. The cell line was obtained in accordance with the ethical guidelines of the supplier. The use of the cell lines was in accordance with the appropriate ethical approvals and was based on the informed consent of the donors.

### Western blotting

Cells were lysed in TNE buffer containing 10 mM Tris-HCl (pH 7.4), 150 mM NaCl, 1 mM EDTA, 1% Nonidet P-40, 50 mM NaF, 2 mM Na_3_VO_4_, 10 μg/mL aprotinin, and 1% phosphatase inhibitor cocktail (Nacalai Tesque, Kyoto, Japan). Proteins from the lysates were separated by sodium dodecyl sulfate-polyacrylamide gel electrophoresis (SDS-PAGE) and transferred onto nitrocellulose membranes. Western blotting was performed using primary antibodies specific for phosphorylated β-catenin (Ser33/37/Thr41) (1:1,000; Cell Signaling Technology, MA, United States), non-phosphorylated (active) β-catenin (1:1,000; Cell Signaling Technology), total β-catenin (1:1,000; Cell Signaling Technology), GLI1 (1:1,000; Cell Signaling Technology), and β-actin (1:1,000; Cell Signaling Technology). Further, membranes were incubated with horseradish peroxidase (HRP)-conjugated secondary antibodies, including goat anti-rabbit (1:2,000; Cell Signaling Technology) and horse anti-mouse (1:2,000; Cell Signaling Technology), as appropriate. Immunoreactive bands were visualized using an enhanced chemiluminescence (ECL) detection system as previously described (Watanabe et al., 2025). Band intensities were quantified using ImageJ software (National Institutes of Health) by measuring the integrated density of each band.

### Statistical analysis

All data are expressed as mean ± standard error of the mean (SEM). Statistical analyses of two groups were conducted by evaluating data normality using the Shapiro–Wilk test. Based on the type of results, either a two-tailed unpaired Student’s t-test (for normally distributed data) or the Mann–Whitney U test (for non-normal data) was employed to assess statistical significance. For comparisons involving three or more groups, one-way analysis of variance (ANOVA) followed by Dunnett’s or Tukey-Kramer’s *post hoc* test, or two-way ANOVA followed by Bonferroni *post hoc* test, was employed. Differences in weighted UniFrac distances were analyzed using pairwise permutational multivariate analysis of variance (PERMANOVA).

## Results

### *Gpr30* is abundantly expressed in the skin and dermal papilla cells

**FIGURE 1:**
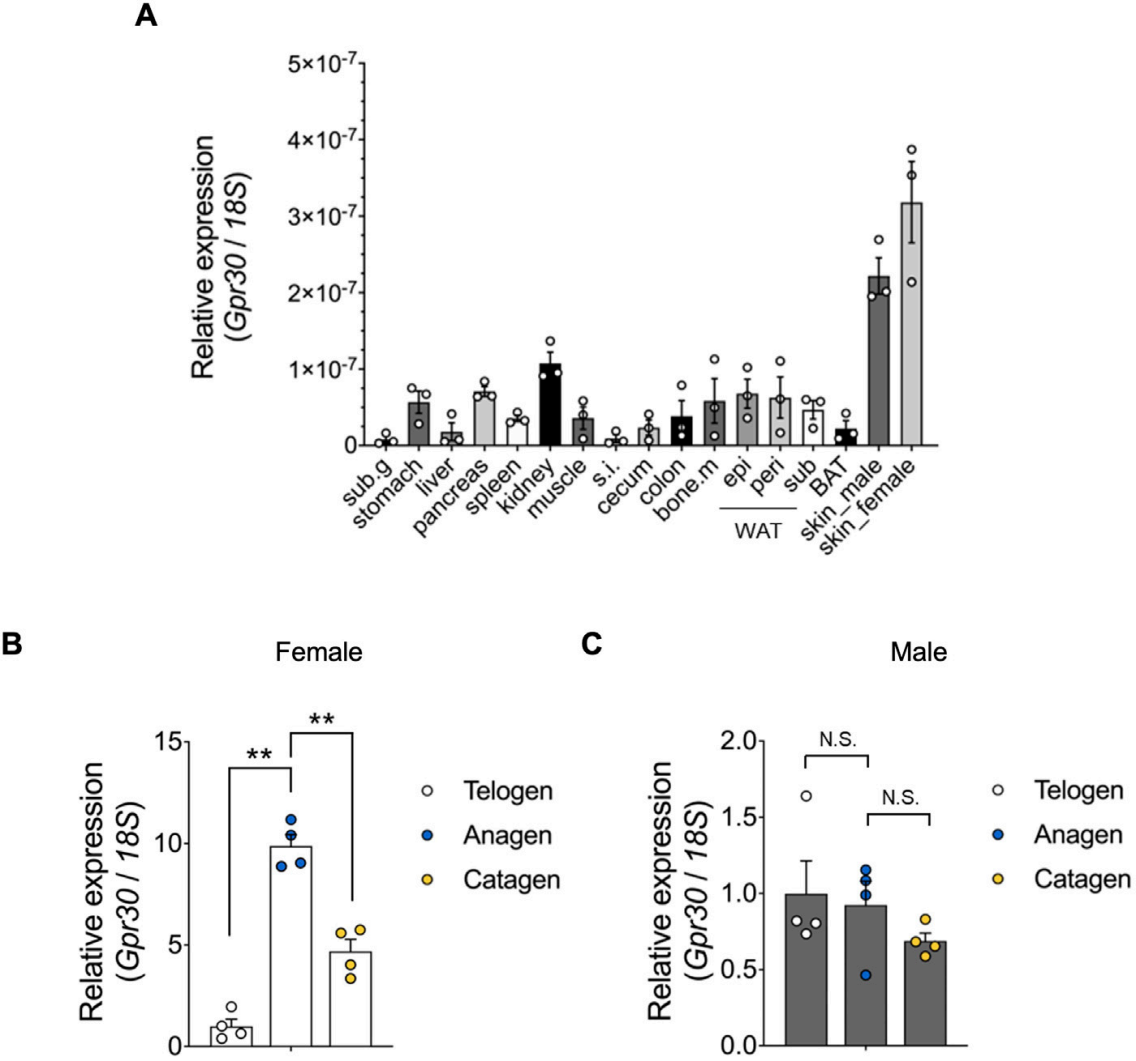
*Gpr30* is abundantly expressed in the skin. **(A)** *Gpr30* mRNA expression in 7-week-old mouse tissues was determined using qRT-PCR (*n* = 3). Sub.g, submandibular gland; s. i., small intestine; bone.m, bone marrow; epi, epididymal; peri, perirenal; sub, subcutaneous; WAT, white adipose tissues; BAT, brown adipose tissues. **(B)** *Gpr30* mRNA expression in female mice during hair follicle cyclic phases determined using qRT-PCR (*n* = 4). **(C)** *Gpr30* mRNA expression in male mice during hair follicle cyclic phases determined using qRT-PCR (*n* = 4). Internal control: *18S* rRNA expression. **p < 0.01 (Tukey’s multiple comparison test). Results are presented as the means ± SE. N.S., not significant.

We examined GPR30 expression across various mouse tissues. Using qRT-PCR, we analyzed *Gpr30* mRNA levels in tissues from post-natal day 49 mice. Although *Gpr30* mRNA was detected in multiple tissues, including white adipose tissues and the stomach, its expression was notably abundant in the skin of both male and female mice (Figure 1A). Hormonal factors such as estrogen and androgen are known to influence hair growth patterns and susceptibility to hair loss (Stenn and Paus, 2001; Grymowicz et al., 2020; Zouboulis et al., 2007; Ohnemus et al., 2006; Conrad and Paus, 2004). Therefore, we further investigated *Gpr30* expression during the hair cycle. The hair cycle consists of three phases: telogen (resting), anagen (active growth), and catagen (regression, when the follicle begins to stop growing) (Fuchs et al., 2001; Clevers et al., 2014; Choi, 2020; Colin-Pierre et al., 2022; Bai et al., 2019). In experiments inducing telogen-to-anagen transition through hair shaving, *Gpr30* mRNA expression was significantly higher during the anagen phase compared to the telogen phase in female mice (Figure 1B). However, no significant difference was observed between telogen and anagen in male mice (Figure 1C). The hair follicle is a complex structure composed of various cell types, including dermal papilla cells, which play a crucial role in regulating hair growth and the hair cycle by signaling to other follicular cells (Fuchs et al., 2001; Clevers et al., 2014; Choi, 2020; Colin-Pierre et al., 2022; Bai et al., 2019). Analysis using the public Gene Expression Library for hair follicles (https://hair-gel.net/) revealed that *Gpr30* is highly expressed in DPCs of mouse hair follicles (Supplementary Figure S1).

### GPR30 stimulation by the selective agonist G-1 promotes hair growth

**FIGURE 2:**
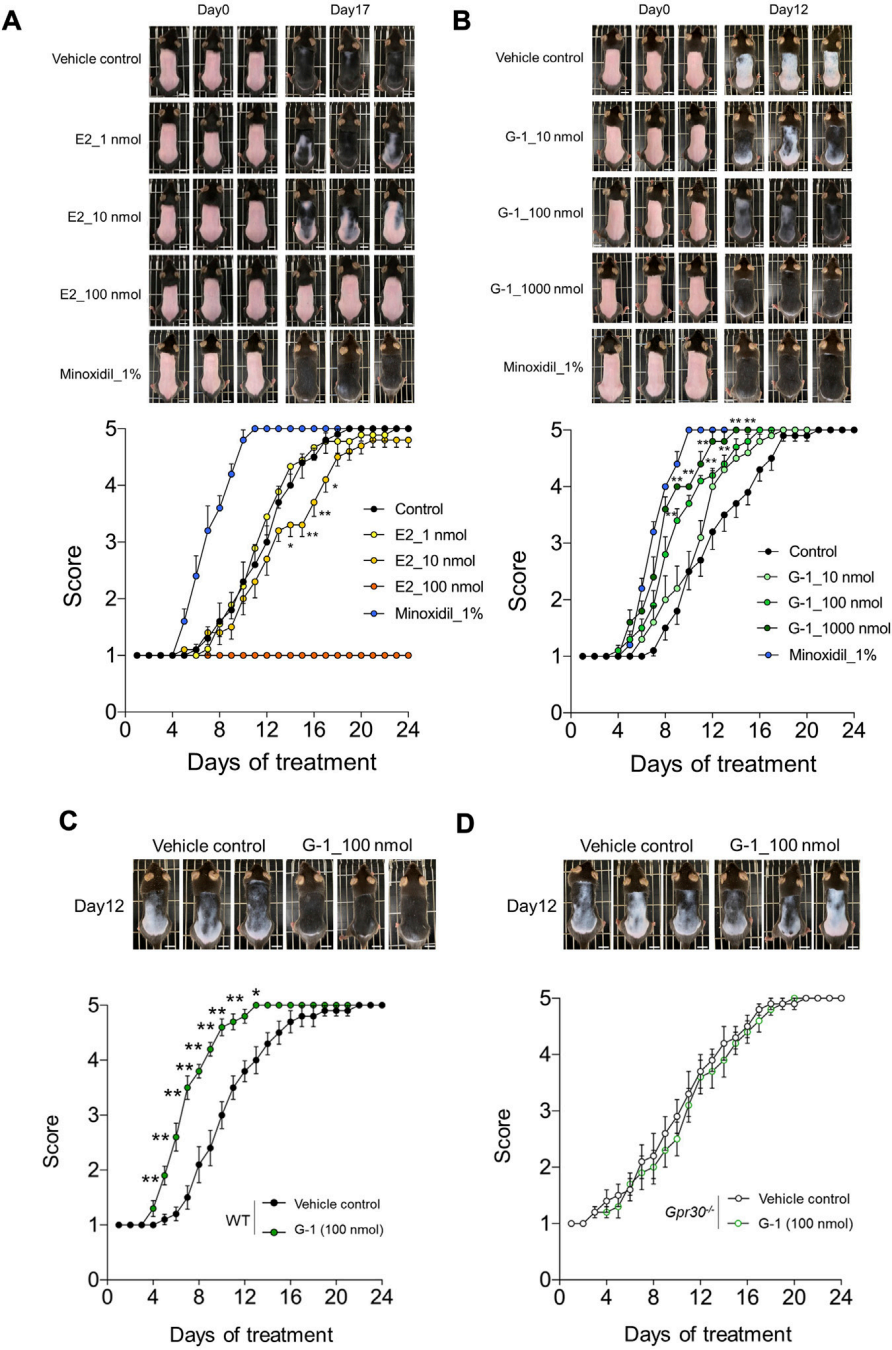
GPR30 selective agonist G-1 promotes hair growth. **(A,B)** Dose-dependent effects of estrogen, G-1, and minoxidil on hair growth in wild-type female mice (A, *n* = 3–10; B, *n* = 5–10). Scale bar = 1 cm. **(C,D)** Effects of G-1 on hair growth in wild-type female mice (C, *n* = 10) and *Gpr30*-deficient female mice (D, *n* = 10). Scale bar = 1 cm. **p < 0.01, *p < 0.05 (two-way ANOVA with the Bonferroni *post hoc* test). Representative photographs were taken on Day 1 and Day 17 post-estrogen treatment, and on Day 12 following treatment with G-1 or minoxidil. These experiments were performed using independent application trial from three biological replicates. Results are presented as means ± SE.

To evaluate the *in vivo* effects of GPR30 on hair growth, we used *Gpr30*-deficient mice (Supplementary Figures S2A–C). In C57BL/6 mice, hair growth was visually assessed by observing changes in skin color, which transitioned from pink to black as the hair cycle progresses from telogen to anagen. In estradiol-treated female mice, the transition to black skin was dose-dependently suppressed compared to control mice, and the dorsal skin color score was significantly lower (Figure 2A). In contrast, G-1 treatment dose-dependently enhanced the transition to black skin, with significantly higher dorsal skin color scores compared to controls (Figure 2B). Similarly, minoxidil, known to promote hair growth (Messenger and Rundegren, 2004) and markedly enhances black pigmentation in skin (Figures 2A,B). In contrast, in G-1-treated male mice, the transition to black skin was comparable to that in control mice, and the dorsal skin color score did not change (Supplementary Figure S3). Additionally, the increased transition from pink to black and the elevated dorsal skin color scores observed in wild-type female mice treated with G-1 (100 nmol) (Figure 2C) were completely abolished in *Gpr30*-deficient female mice (Figure 2D). These findings demonstrate that GPR30 activation by G-1 promotes hair growth by stimulating the transition to the anagen phase in female mice.

### G-1 stimulation activates Wnt signaling in the skin

**FIGURE 3:**
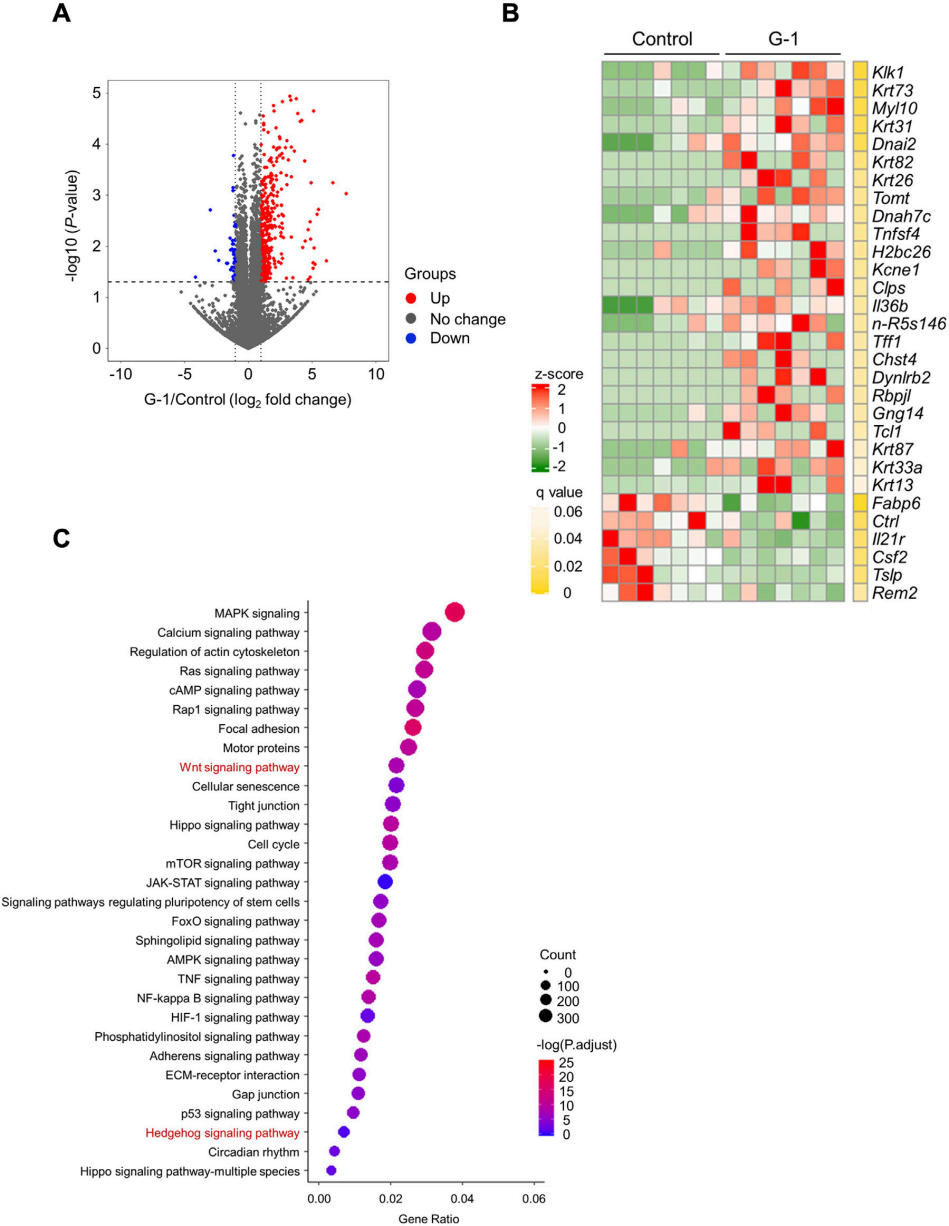
RNA sequencing analysis shows activation of Wnt/Hedgehog signaling by G-1 stimulation in the skin. **(A)** Volcano plot showing the significance and magnitude of differences in the relative abundances of variable genes in the skin of female mice with or without G-1 treatment (*n* = 7). **(B)** Heat map of the top 30 variable gene profiles in the skin of female mice with or without G-1 treatment (*n* = 7). **(C)** KEGG enrichment analysis related to molecular function in the skin of wild-type female mice with or without G-1 treatment (*n* = 7). *p*-values were adjusted using the false discovery rate (FDR).

**FIGURE 4:**
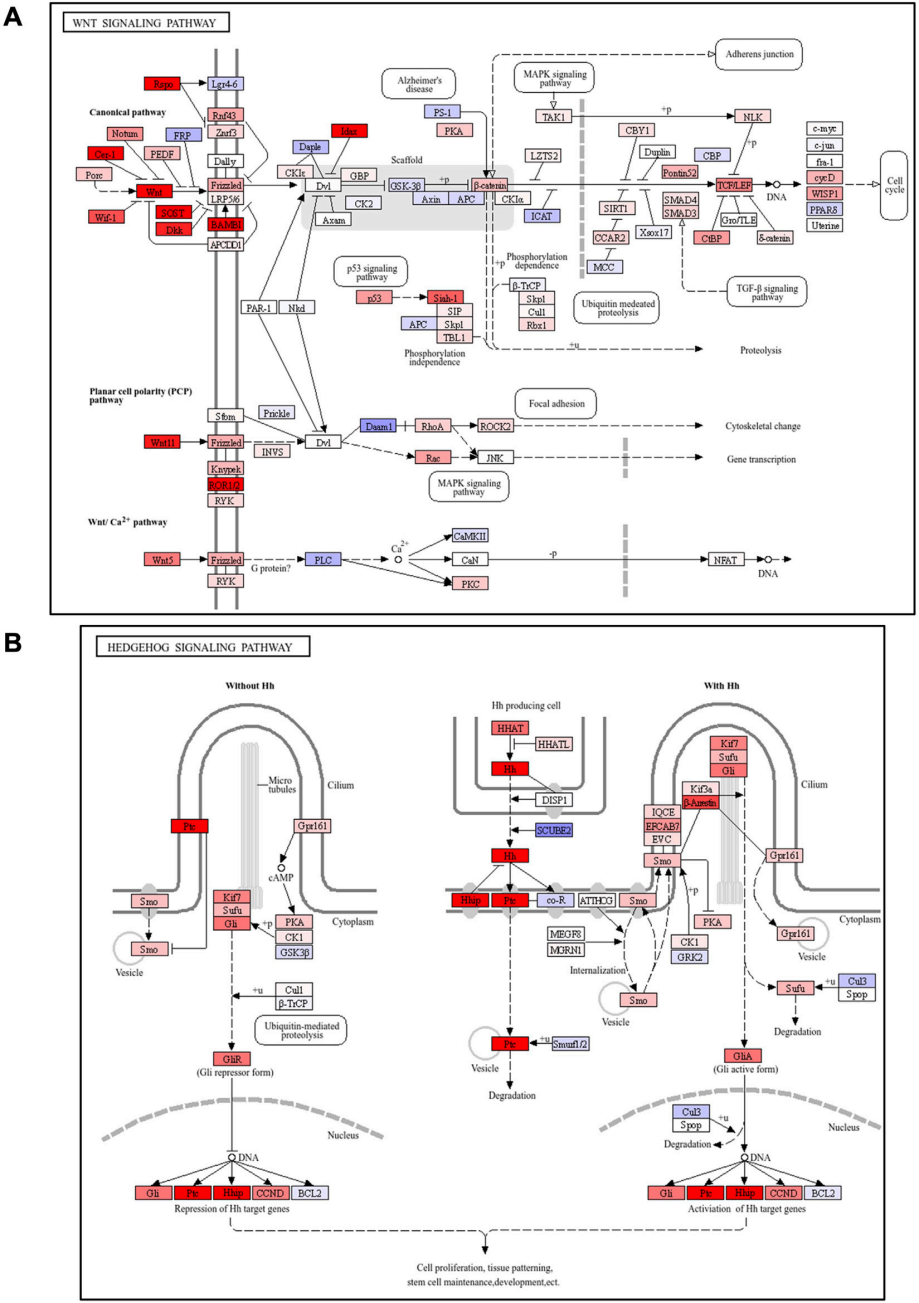
Pathway analysis showed activation of Wnt/Hedgehog signaling following G-1 stimulation in the skin. **(A,B)** KEGG pathway analysis of Wnt or Hedgehog signaling-related factors in the skin of wild-type female mice with or without G-1 treatment (*n* = 7).

We performed RNA sequencing of skin tissues to investigate the effects of G-1-mediated GPR30 activation on gene expression and associated signaling pathways. RNA sequencing analysis revealed significant changes in the expression of 2,144 genes following G-1 treatment in the skin of wild-type female mice (Figure 3A). Among these, seven of the top 30 significantly altered genes were directly related to hair growth (Figure 3B), highlighting the potential role of G-1 in modulating hair follicle activity. Gene Ontology (GO) analysis indicated significant enrichment of the Wnt and Hedgehog signaling pathway in the skin of G-1-treated female mice (Figure 3C). This observation was further supported by Kyoto Encyclopedia of Genes and Genomes (KEGG) pathway analysis, which also demonstrated upregulation of the Wnt and Hedgehog signaling pathways in response to G-1 treatment (Figures 4A,B). Moreover, hair growth-related pathways, such as BMP (a negative regulator of hair growth: suppresses hair follicle stem cell activation and helps maintain the quiescence of stem cells during the telogen phase) (Pujades et al., 2006) and FGF signaling (involved in epidermal-mesenchymal interactions that are crucial for follicle regeneration) (Jacques et al., 2012), were not associated with G-1 treatment (Supplementary Figure S4). These findings suggest that G-1 stimulation in wild-type female mice enhances the expression of genes associated with hair growth and significantly promotes activation of the Wnt and Hedgehog signaling pathway, a critical regulator of hair follicle development and regeneration.

### G-1 stimulation promotes GPR30-mediated Wnt-signaling in the skin

**FIGURE 5:**
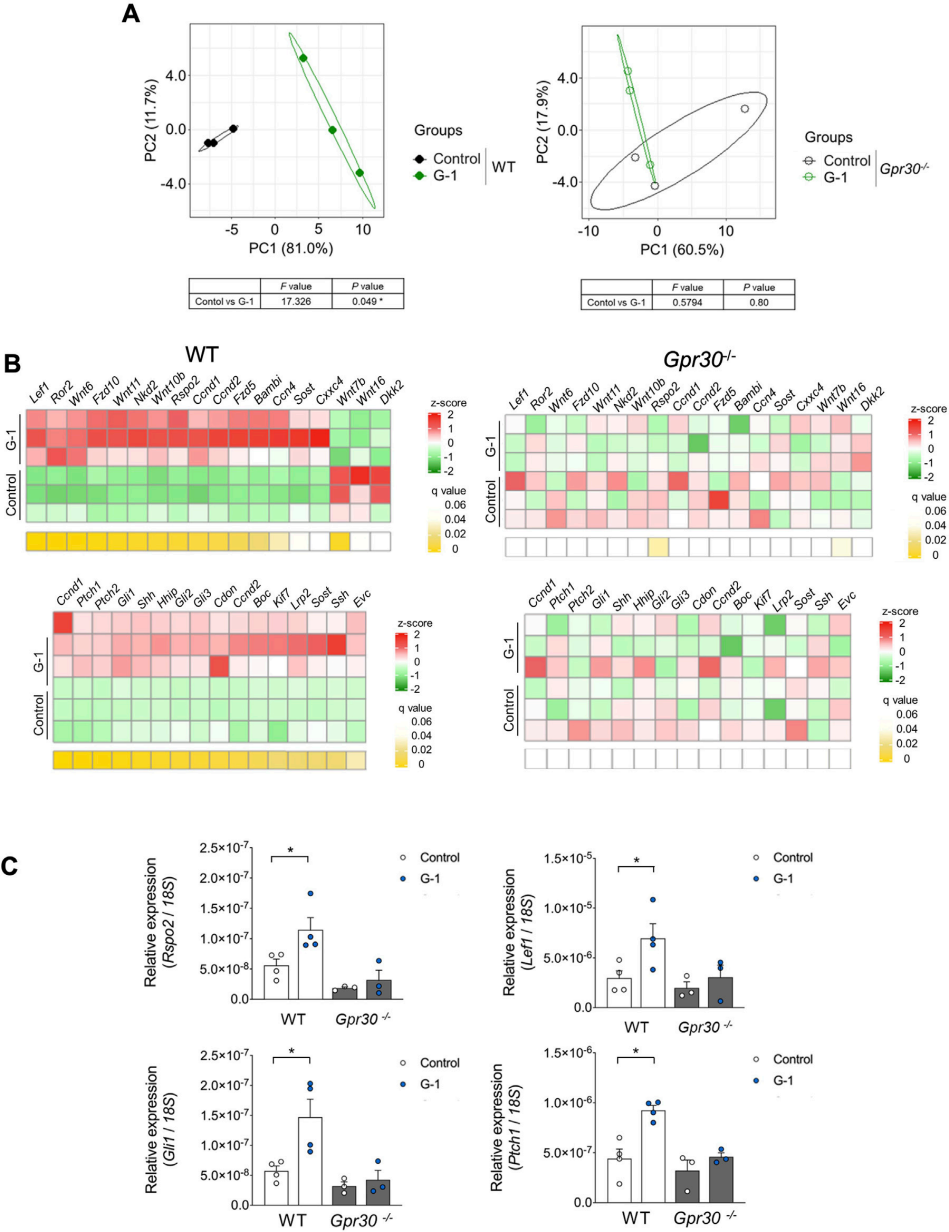
G-1 promotes Wnt/Hedgehog signaling via GPR30 in the skin. **(A)** Beta diversity as shown via principal component analysis (PCA) based on genes from KEGG in the skin of wild-type and *Gpr30*-deficient female mice with or without G-1 treatment (*n* = 3). Compositional similarity was compared using PERMANOVA. **(B)** Heat map of Wnt/Hedgehog-related gene profiles in the skin of wild-type and *Gpr30*-deficient female mice with G-1 treatment (*n* = 3). *p*-values were adjusted using the false discovery rate (FDR). **(C)** mRNA expression levels of Wnt/Hedgehog signaling-related genes in the skin of wild-type and *Gpr30*-deficient female mice measured by qRT-PCR (*n* = 3–4). Internal controls: *18S* rRNA expression. *p < 0.05 (Mann–Whitney U test). All data are presented as the means ± SE.

**FIGURE 6:**
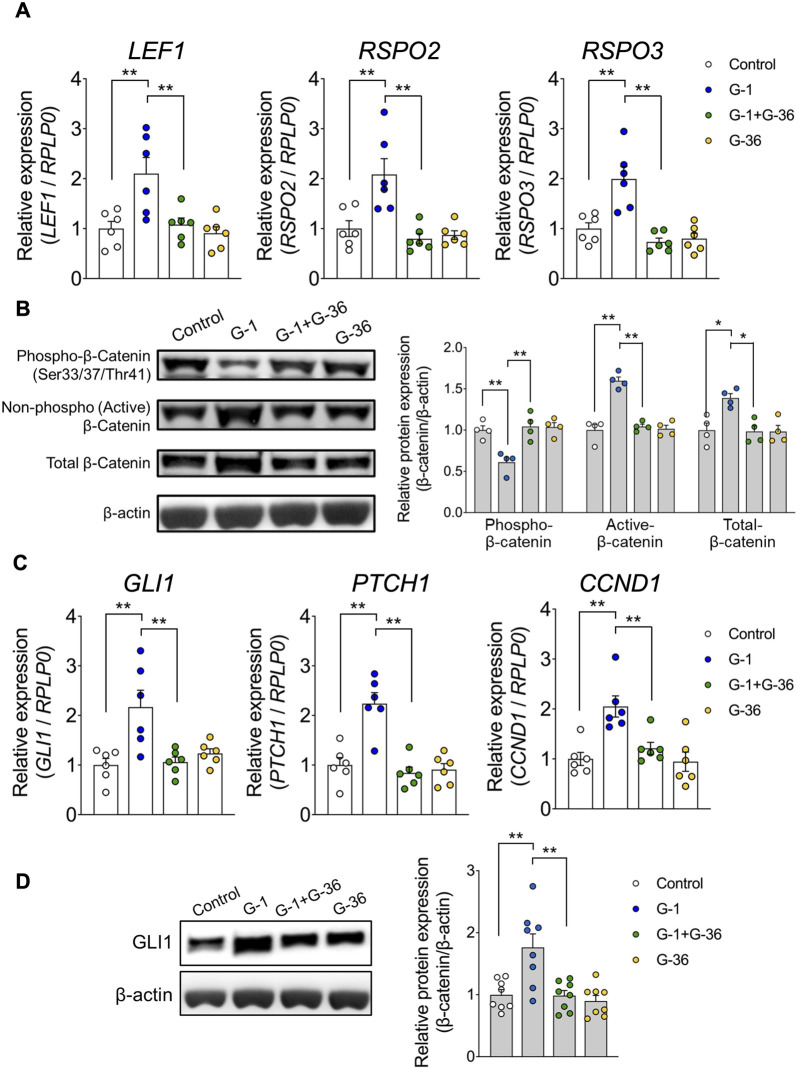
G-1 promotes Wnt/Hedgehog-signaling in the human primary DPCs. **(A)** mRNA expression levels of Wnt signaling-related genes in human hair follicle dermal papilla cells (hDPCs) stimulated with G-1 with or without G-36 measured by qRT-PCR (*n* = 6). Internal controls: *RPLP0* expression. **(B)** Activation of the Wnt signaling-related protein β-Catenin in hDPCs stimulated with G-1, with or without G-36, measured using Western blotting (*n* = 4). Internal controls: β-actin expression. **(C)** mRNA expression levels of Hedgehog signaling-related genes in hDPCs stimulated with G-1, with or without G-36, measured using qRT-PCR (*n* = 6). Internal controls: *RPLP0* expression. **(D)** Expression levels of Hedgehog signaling-related proteins in hDPCs stimulated with G-1, measured using Western blotting (*n* = 8). Internal controls: β-actin expression. *p < 0.05, **p < 0.01 (Tukey–Kramer's post hoc test). All data are presented as the means ± SE.

To further investigate the effects of G-1 treatment, we analyzed changes in gene expression using PCA. PCA revealed significant differences along PC 1 and PC 2 between G-1 treated groups and control groups in wild-type female mice. These differences were absent in *Gpr30*-deficient female mice (Figure 5A). Similarly, a heatmap of Wnt and Hedgehog signaling-related genes showed significant gene expression in G-1-treated wild-type female mice compared to untreated controls, whereas no changes were observed in *Gpr30*-deficient female mice (Figure 5B). These results indicate that G-1 mediated GPR30 activation interacts with pathways like Wnt/β-catenin and Hedgehog to regulate hair follicle stem cell activity and the transition between hair cycle phases. To explore the direct relationship between Wnt/Hedgehog signaling and G-1 stimulation, we analyzed the expression of specific Wnt and Hedgehog signaling-related genes. The expression of *Rspo2* (a secreted protein that enhances Wnt receptor sensitivity), *Lef1* (a transcription factor involved in the canonical Wnt/β-catenin signaling pathway), *Gli1* (transcriptional activator in Hedgehog signaling), *Ptch1* (Hedgehog receptor), and *Ccnd1* (cell cycle regulator and Hedgehog target) (Fuchs et al., 2001; Clevers et al., 2014; Choi, 2020; Colin-Pierre et al., 2022; Bai et al., 2019) significantly increased in the skin of G-1-treated wild-type female mice compared to control wild-type female mice (Figure 5C). Moreover, in human primary DPCs, *RSPO2*, *RSPO3* and *LEF1* mRNA levels were significantly elevated following G-1 treatment, but these effects were abolished by treatment with the GPR30 antagonist G-36 (Dennis et al., 2011; Figure 6A). Additionally, β-catenin activation was promoted by G-1 treatment but was abolished following G-36 treatment (Figure 6B). Moreover, *GLI1*, *PTCH1, CCND1* mRNA levels and GLI1 protein levels were significantly elevated following G-1 treatment, and the effects were abolished by G-36 treatment (Figures 6C,D). These results indicate that GPR30 activation by G-1 promotes hair growth by activating the Wnt and Hedgehog signaling pathway.

## Discussion

**FIGURE 7:**
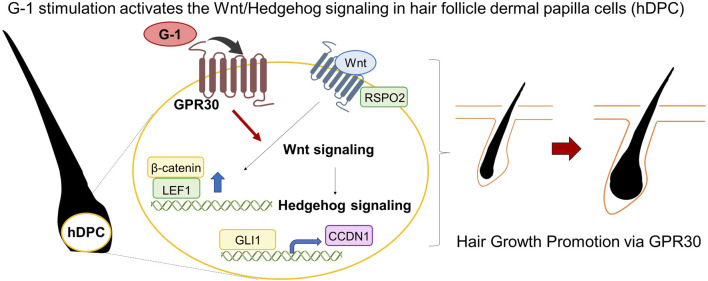
Schematic representation. G-1 stimulation activates the Wnt/Hedgehog signaling.

This study demonstrated that GPR30 is highly expressed in mouse skin, particularly during the anagen phase of the female hair follicle cycle, suggesting its role in regulating hair growth. Activation of GPR30 using the selective agonist G-1 in both female mouse skin and human dermal papilla cells significantly enhanced Wnt/Hedgehog signaling, a crucial pathway for hair growth (Figure 7). Further, these effects were absent in *Gpr30*-deficient female mice and those treated with an GPR30 antagonist, confirming the indispensable role of GPR30 in estrogen-mediated regulation of hair follicle function.

Our *in vivo* experiments revealed that G-1 treatment effectively promoted hair regrowth, whereas high-dose estrogen treatment suppressed it. Estrogen activates the Wnt/β-catenin signaling pathway, which is critical for hair follicle development and regeneration (Wang et al., 2010). Activation of this pathway promotes the proliferation of DPCs and initiates the anagen phase (Kishimoto et al., 2000), whereas excess estrogen disrupts the normal hair cycle and contributes to hair thinning or shedding (Ohnemus et al., 2012). Additionally, estrogen has inhibitory effects, such as reducing hair density or the growth rate of body hair (Thornton, 2002). ERα and ERβ activates pro-growth molecular pathways including Wnt/β-catenin, IGF-1, and VEGF, which support hair follicle proliferation and vascularization in dermal papilla cells (Enshell-Seijffers et al., 2010). The difference between G-1 and estrogen may suggest that GPR30 activation functions independently compared with nuclear estrogen receptors and other estrogen-related mechanisms. Additionally, *Gpr30* mRNA expression was significantly increased during the anagen phase of the hair cycle in female mice but not in male mice, despite comparable baseline expression levels in the skin of both sexes, and hair growth was not promoted by G-1 treatment in male mice. Future studies are needed to elucidate the molecular mechanisms underlying GPR30’s role in hair growth, specifically focusing on the distinct contributions of GPR30, ERα, ERβ, and other estrogen signaling pathways. These findings may help clarify the relationship between sex differences and estrogen levels during the hair cycle.

Although this study showed that GPR30 activation by G-1 promotes hair growth, it remains unclear whether estrogen directly influences hair growth through GPR30. Moreover, our findings demonstrate that the promotion of hair growth and Wnt and Hedgehog signaling by G-1 stimulation was abolished in *Gpr30*-deficient mice and following treatment with a GPR30 antagonist. These results provide novel insights into the role of GPR30 in hair follicle biology. Further studies are required to investigate the intracellular GPCR signaling pathways mediated by GPR30 and their interactions with Wnt and Hedgehog signaling.

Although the hair growth-promoting effect of G-1 was not stronger than that of minoxidil, it exhibited sex-specific efficacy, promoting hair growth in female mice but not in males. GPR30 activation following G-1 treatment activated the Wnt and Hedgehog signaling pathways in hair follicle cells, both of which are critical for hair follicle activation and regeneration. These findings suggest that the hair growth-promoting mechanism of G-1 differs from that of minoxidil, which acts via vasodilation and potassium channel activation rather than direct modulation of hair follicle-related signaling pathways. Given the distinct molecular mechanisms of G-1 and minoxidil, they may exert synergistic or additive effects when used in combination. Furthermore, the sex-specific activity of G-1 presents the possibility of developing sex-tailored hair loss treatments, particularly for female patients who may benefit from estrogen-targeted therapies. Future studies are necessary to explore optimal dosing regimens, long-term efficacy, and safety profiles of combination therapies involving G-1 and existing hair growth agents, such as minoxidil.

Our study also showed that G-1 stimulation promoted Wnt and Hedgehog signaling in primary cultures of human hair follicle dermal papilla cells. These results suggest that GPR30 activation directly affects the skin rather than indirectly influencing hair growth through other tissues. However, further experiments using tissue-specific *Gpr30*-deficient mice are necessary to confirm the precise role of GPR30 in hair follicles. Additionally, because our *in vivo* experiments were conducted primarily in murine models, the translational relevance of these findings to human physiology requires further exploration. While GPR30 agonists, such as G-1, show promise as therapeutic targets, their safety and long-term effects remain unclear, particularly those related to GPR30 expression in multiple tissues. Systemic activation may cause off-target effects, including hormonal imbalances or dermatological reactions. Furthermore, chronic stimulation of pathways such as Wnt and Hedgehog carries potential risks, such as abnormal cell proliferation. The lack of data on tissue-specific actions and long-term outcomes limits the current understanding of the therapeutic relevance of G-1. Additional studies are required to address these concerns and evaluate the feasibility of translating murine results to human applications. Understanding sex-based differences and hormonal influences will also be crucial in optimizing future GPR30-targeted therapies.

## Conclusion

This study highlights GPR30 as a promising therapeutic target for estrogen-responsive conditions, including hair loss disorders. The ability of GPR30 activation to enhance Wnt/Hedgehog signaling and promote anagen transition in hair follicles offers novel opportunities for developing targeted therapies.

## Data Availability

All data generated or analyzed during this study are included in the article/Supplementary Material files, or are available from the corresponding authors upon reasonable request. The RNA sequencing dataset has been deposited in the DNA Data Bank of Japan with accession numbers DRA020109, DRA021397, E-GEAD-908, and E-GEAD-1078, available at: https://ddbj.nig.ac.jp/public/ddbj_database/gea/experiment/E-GEAD-000/E-GEAD-908/; https://ddbj.nig.ac.jp/public/ddbj_database/gea/experiment/E-GEAD-1000/E-GEAD-1078/; https://ddbj.nig.ac.jp/search/entry/sra-submission/DRA020109; https://ddbj.nig.ac.jp/search/entry/sra-submission/DRA021397. All data relevant to this study were deposited in the Dryad database (DOI:10.5061/dryad.xwdbrv1qj).

## Supplementary material

SUPPLEMENTARY FIGURE S1
*Gpr30* mRNA expression profiles in hair cells. *Gpr30* mRNA expression was analyzed in hair cells of postnatal day 5 mice (*n* = 2) using the database (http://www.hair-gel.net/). All data are presented as the means. These experiments were performed with independent cell populations isolated from fluorescent reporter mouse lines across three biological replicates. Epi, epidermis; ORS, outer root sheath; Mx, matrix; TAC, transit amplifying progenitors; Mc, melanocytes; DF, dermal fibroblasts; DP, dermal papilla; Neg, mixed negative cells; HF-SC, hair follicle stem cell precursors; HF-ORS, hair follicle outer root sheath; ZZ-DP, zigzag dermal papilla; G/AA-DP, guard/awl and auchene dermal papilla; G-DP, guard dermal papilla; AA/ZZ-DP, awl and auchene/ zigzag dermal papilla.

SUPPLEMENTARY FIGURE S2 Schematic overview of the structure and expression of the *Gpr30* gene. **(A)** *Gpr30* gene-knockout (*Gpr30* ^*−/−*^) mice were generated using the CRISPR/Cas9 system in wild-type C57BL/6 zygotes. Coding exon regions are depicted as black boxes and labeled accordingly. The guide RNA (gRNA) and protospacer adjacent motif (PAM) are represented by light blue and purple boxes, respectively. **(B)** Mouse genotypes were identified by PCR using specific primers to differentiate between wild-type and mutant alleles of *Gpr30*. **(C)** Expression of the *Gpr30* gene was analyzed in skin samples (*n* = 4 tissues). Internal control: *18S* rRNA expression. All data are presented as the means ± SE.

SUPPLEMENTARY FIGURE S3 GPR30 selective agonist G-1 does not promote hair growth in male mice. Effects of G-1 (100 nmol) on hair growth in wild-type male mice (*n* = 10). Scale bar = 1 cm. (two-way ANOVA with the Bonferroni post hoc test). Representative photographs were taken on Days 1 and 17 following G-1 treatment. These experiments were performed using independent application trials from three biological replicates. Results are presented as means ± SE.

SUPPLEMENTARY FIGURE S4 RNA sequencing analysis showed irrelevant BMP and FGF signaling following G-1 stimulation in the skin. Heat map of BMP and FGF-related gene profiles in the skin of female mice with or without G-1 treatment (*n* = 7). *p*-values were adjusted using the false discovery rate (FDR).
