# Supplementary figures and images for: The GPR30 agonist G-1 promotes hair growth via Wnt/Hedgehog signaling in mice
Source: Front Pharmacol. 2025 Jul 11;16:1570922. doi: 10.3389/fphar.2025.1570922 (PMC12289589; doi:10.3389/fphar.2025.1570922)

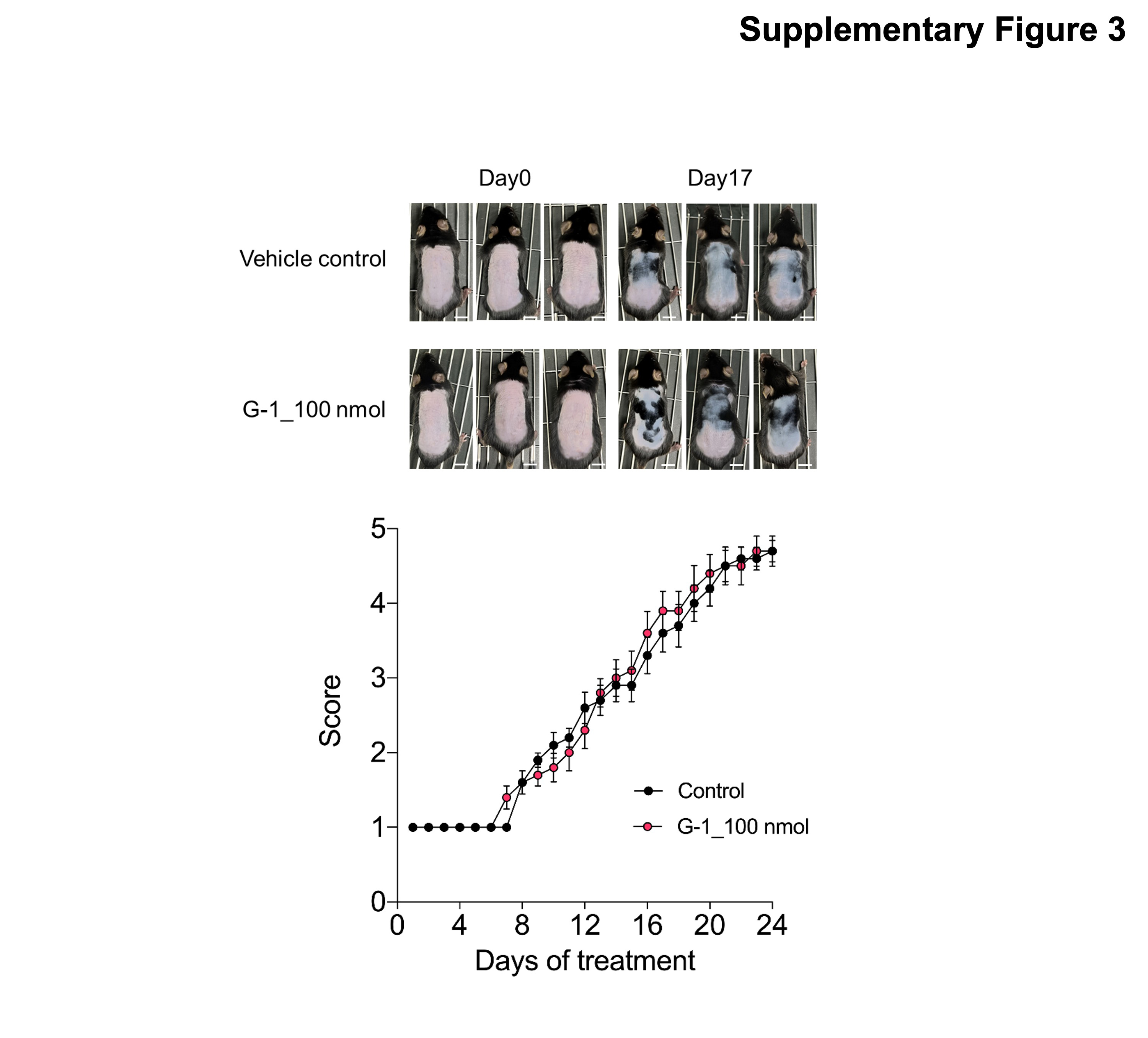

Supplement: Supplementary file 1 [file Image3.jpeg]

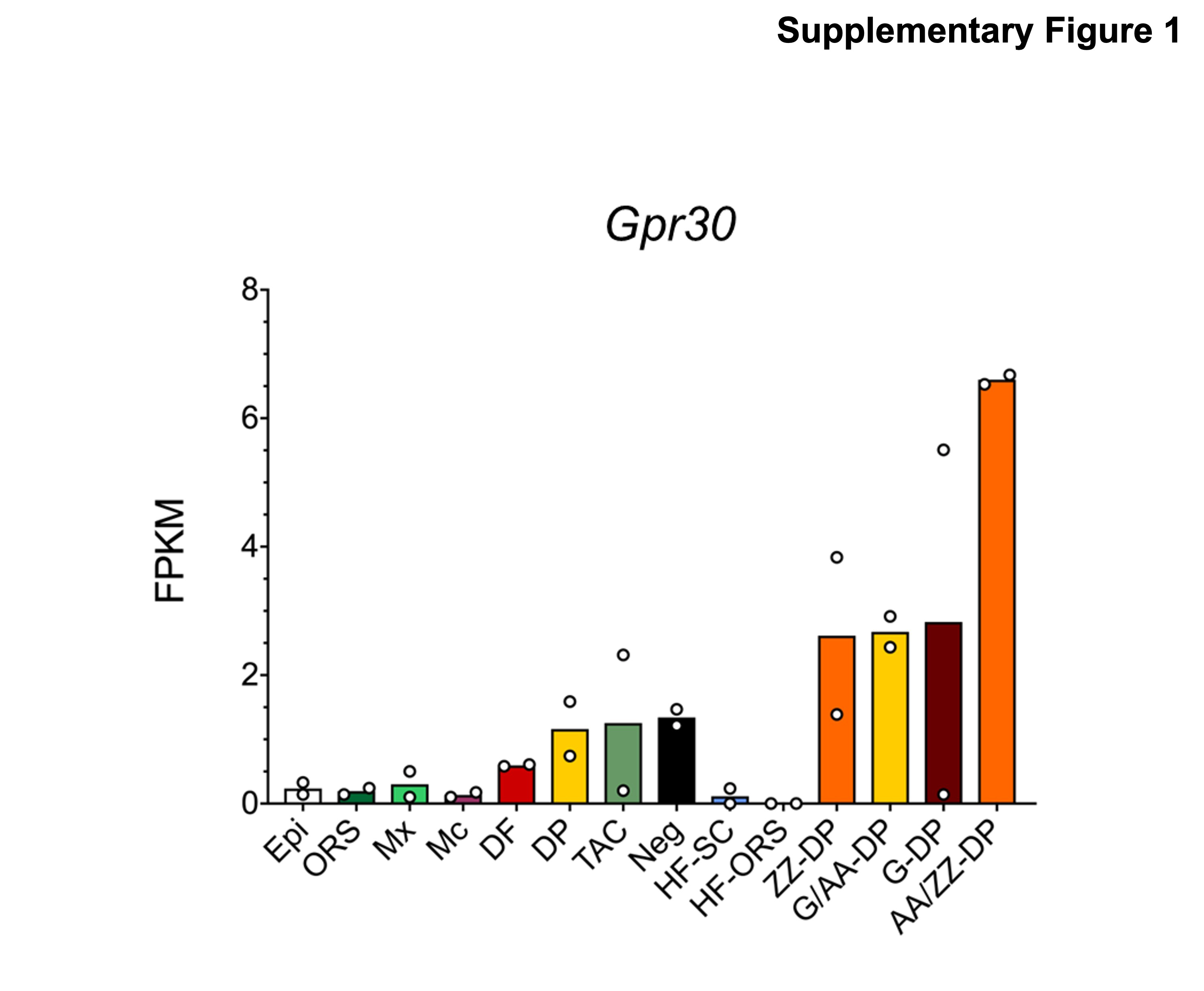

Supplement: Supplementary file 2 [file Image1.jpeg]

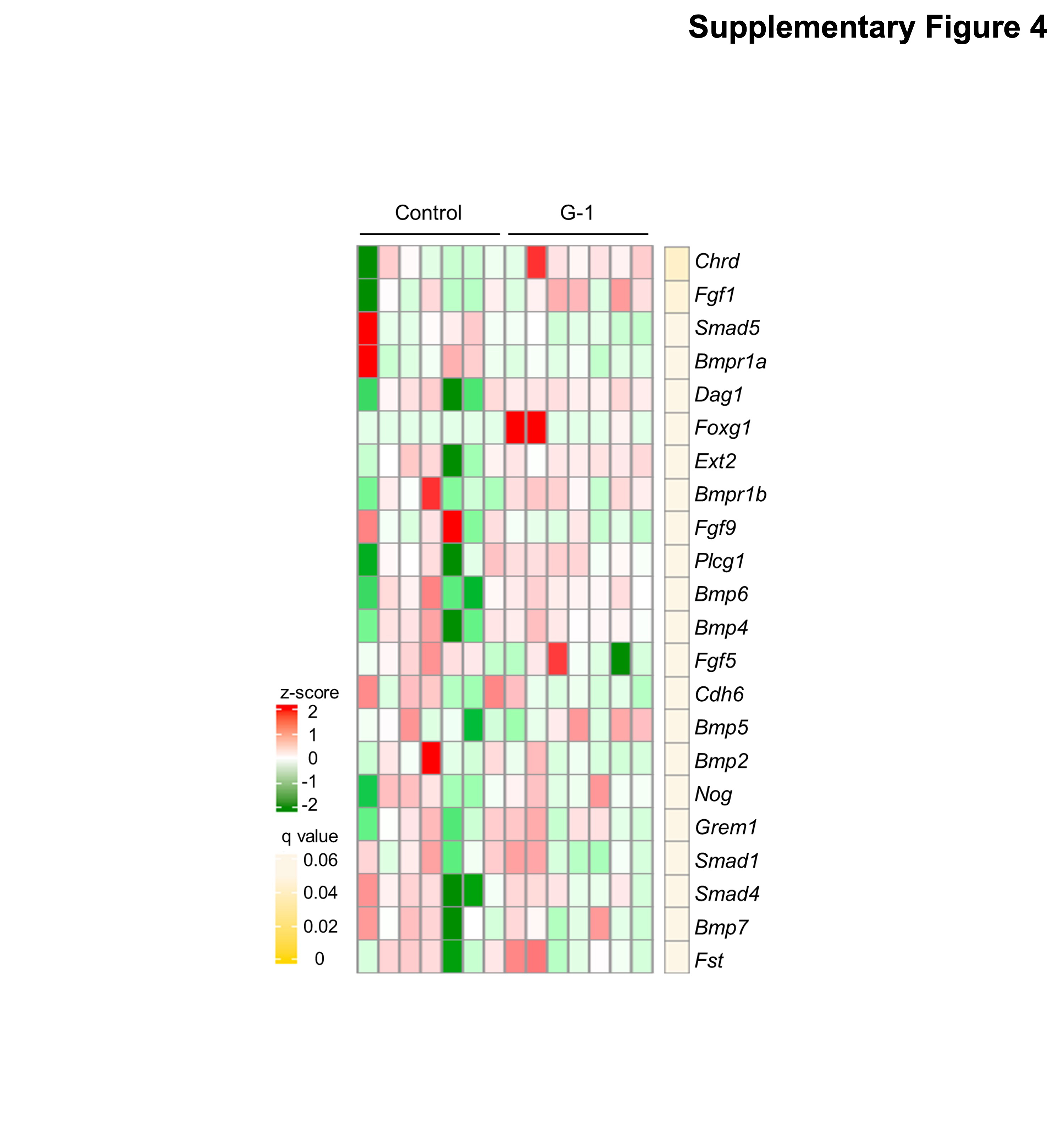

Supplement: Supplementary file 3 [file Image4.jpeg]

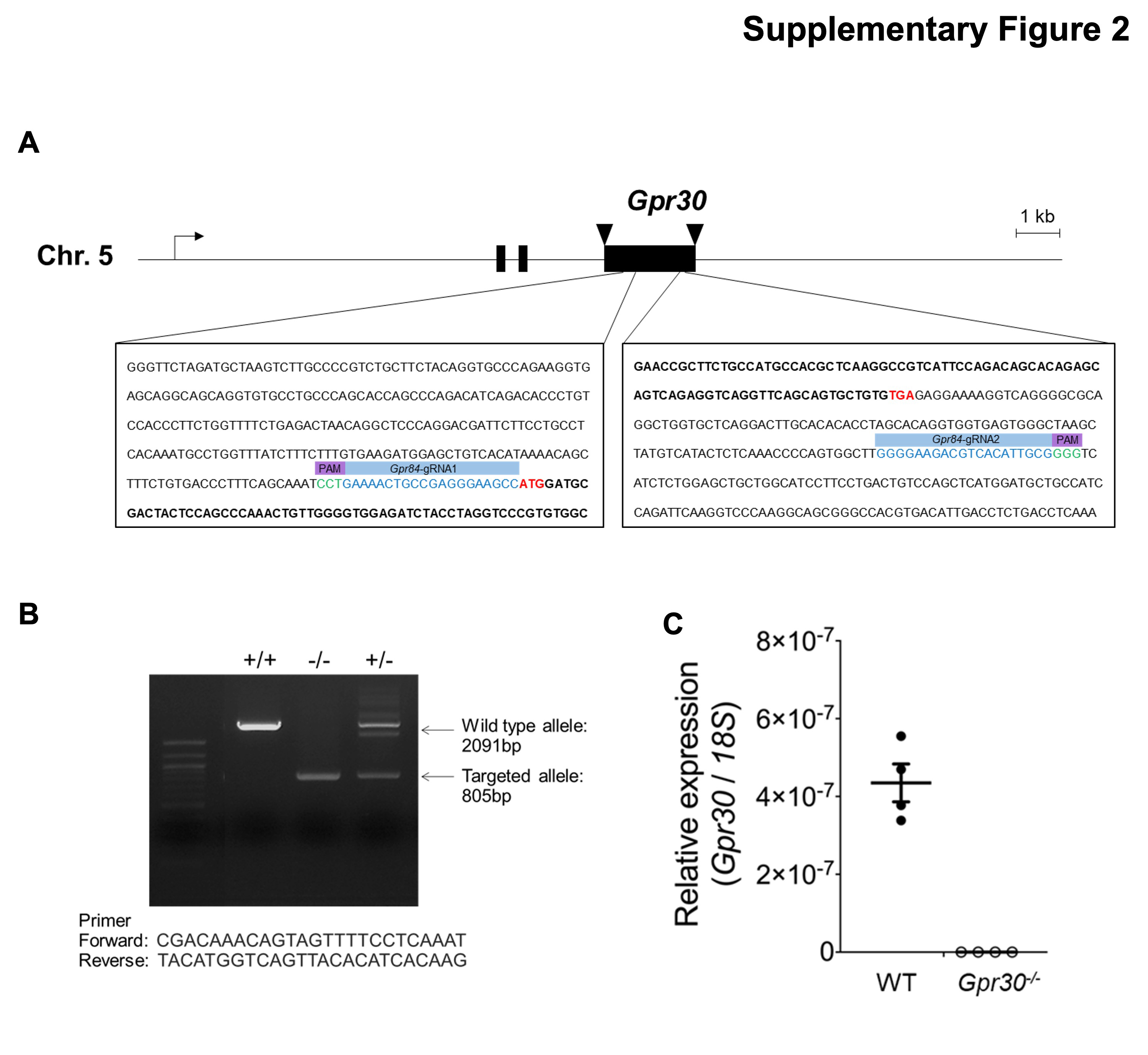

Supplement: Supplementary file 4 [file Image2.jpeg]
